# Dual Function of Wnt Signaling during Neuronal Differentiation of Mouse Embryonic Stem Cells

**DOI:** 10.1155/2015/459301

**Published:** 2015-04-05

**Authors:** Hanjun Kim, Sewoon Kim, Yonghee Song, Wantae Kim, Qi-Long Ying, Eek-hoon Jho

**Affiliations:** ^1^Department of Life Science, University of Seoul, 163 Seoulsiripdaero, Dongdaemun-gu, Seoul 130-743, Republic of Korea; ^2^Eli and Edythe Broad Center for Regenerative Medicine and Stem Cell Research at USC, Keck School of Medicine, University of Southern California, Los Angeles, CA 90033, USA

## Abstract

Activation of Wnt signaling enhances self-renewal of mouse embryonic and neural stem/progenitor cells. In contrast, undifferentiated ES cells show a very low level of endogenous Wnt signaling, and ectopic activation of Wnt signaling has been shown to block neuronal differentiation. Therefore, it remains unclear whether or not endogenous Wnt/*β*-catenin signaling is necessary for self-renewal or neuronal differentiation of ES cells. To investigate this, we examined the expression profiles of Wnt signaling components. Expression levels of Wnts known to induce *β*-catenin were very low in undifferentiated ES cells. Stable ES cell lines which can monitor endogenous activity of Wnt/*β*-catenin signaling suggest that Wnt signaling was very low in undifferentiated ES cells, whereas it increased during embryonic body formation or neuronal differentiation. Interestingly, application of small molecules which can positively (BIO, GSK3*β* inhibitor) or negatively (IWR-1-endo, Axin stabilizer) control Wnt/*β*-catenin signaling suggests that activation of that signaling at different time periods had differential effects on neuronal differentiation of 46C ES cells. Further, ChIP analysis suggested that *β*-catenin/TCF1 complex directly regulated the expression of *Sox1* during neuronal differentiation. Overall, our data suggest that Wnt/*β*-catenin signaling plays differential roles at different time points of neuronal differentiation.

## 1. Introduction

Embryonic stem (ES) cells are pluripotent and self-renewing cells derived from the inner cell mass of preimplantation blastocysts. These cells can differentiate into the following three germ layers: the ectoderm, mesoderm, and endoderm. Due to these characteristics, ES cells are considered useful tools in both research and regenerative medicine. Therefore, it is important to understand how stem cells maintain their self-renewal capacity and differentiate into each specific lineage [1–3]. However, the molecular mechanisms of ES cell self-renewal and differentiation remain poorly understood.

ES cells can be differentiated into neuronal cell lineage* in vitro*. Generally, for differentiation into neuronal cells, ES cells are aggregated to form embryoid bodies (EBs) in suspension culture for 4 days and treated with retinoic acid (RA) [4]. However, it is difficult to manipulate a specific lineage as RA treatment has been shown to induce other cell lineages. To avoid these problems, a chemically defined medium for neuronal differentiation was developed [5, 6]. Using N2B27 medium, 46C cells (mouse ES cells containing Sox1 promoter-driven EGFP) can be reproducibly differentiated into a neuronal lineage without EB formation or treatment with RA, and thereby it has been used to examine the effects of gene manipulation such as depletion or ectopic expression on neuronal differentiation [5, 6].

Wnts are secreted signaling proteins that regulate a variety of developmental processes, including cell growth, motility, and differentiation [7]. Wnt/*β*-catenin signaling is conserved in worms, flies, fish, frogs, mice, and humans [8]. In the absence of Wnt, *β*-catenin forms a “destruction complex” along with the scaffold protein Axin, the tumor suppressor gene product APC, and GSK-3*β* that is phosphorylated by casein kinases (CK1) and glycogen synthase kinase 3*β* (GSK-3*β*). Phosphorylation of *β*-catenin triggers its ubiquitination by *β*-TrCP, followed by proteasomal degradation [9, 10]. However, when Wnt binds to its receptor Frizzled and coreceptor LRP5/6, the cytoplasmic component dishevelled blocks *β*-catenin degradation, leading to the accumulation of *β*-catenin in the cytoplasm. Stabilized *β*-catenin then enters the nucleus where it interacts with TCF/LEF [11–13] and activates the expression of target genes such as* C-Myc*,* Cyclin D1*,* Brachury*, and* Twin*.

Wnt/*β*-catenin signaling has been shown to play important roles in ES cell self-renewal and differentiation [14–17]. Activation of Wnt/*β*-catenin signaling prevents differentiation of human and mouse ES cells. For example, activation of Wnt signaling by GSK3*β* inhibitor maintains self-renewal of human and mouse ES cells, and Wnt/*β*-catenin signaling upregulates Stat3 activity and prevents differentiation of mouse ES cells [18, 19]. Furthermore, TCF3, a terminal transcription factor in Wnt signaling, represses Nanog and delays differentiation of ES cells [20]. However, other evidence has shown that activation of Wnt/*β*-catenin signaling by Wnt3a recombinants induces cell differentiation as well as proliferation [21]. Wnt/*β*-catenin signaling is also involved in regulating the neuronal differentiation of ES cells [22]. On the other hand, it has been shown that the Wnt antagonist sFRP2 induces neuronal differentiation [23]. Therefore, the roles of endogenous Wnt/*β*-catenin signaling and its components in the self-renewal and neuronal differentiation of ES cells remain controversial.

In this study, we examined the endogenous level of Wnt/*β*-catenin signaling components during neuronal differentiation. The data using reporter ES cell lines representing endogenous Wnt/*β*-catenin signaling activity showed that Wnt/*β*-catenin signaling increased during neuronal differentiation. Interestingly, Wnt/*β*-catenin signaling increased until formation of neuronal precursor cells; however, it was reduced during later periods of neuronal differentiation. Differential regulation of neuronal differentiation by the treatment of activator or inhibitor of Wnt/*β*-catenin signaling suggested that Wnt signaling plays dual roles during neuronal differentiation.

## 2. Materials and Methods

### 2.1. Cell Culture

E14 and 46C ES cells (ES cell line in which EGFP is substituted into the ORF of the* Sox1* gene, a reporter for differentiation into neural precursor cells) were cultured in mouse ES (mES) cell medium (DMEM (Gibco) supplemented with 15% FBS, 2 mM GlutaMAX (Gibco), MEM nonessential amino acids, *β*-mercaptoethanol (Gibco), tylosin, and 1% Pen/Strep (Gibco)) supplemented with LIF (ESGRO, Chemicon) on 0.2% gelatin-coated dishes. Cells were maintained at 37°C in a humidified atmosphere of 5% CO_2_.

### 2.2. Neuronal Differentiation

To induce neuronal differentiation, undifferentiated 46C ES cells were trypsinized and neutralized with serum-containing medium. ES cells were dissociated in N2B27 medium (DMEM/F12 (Gibco), Neurobasal medium (Gibco), N2 supplement (Invitrogen), B27 supplement (Invitrogen), 1 mM GlutaMAX (Gibco), 0.1 M *β*-mercaptoethanol (Gibco), and 1% Pen/Strep (Gibco)) and then plated on a 0.2% gelatin-coated tissue culture dish (Falcon) containing DMSO (SIGMA), 0.75 *μ*M MeBIO (Calbiochem), 0.75 *μ*M BIO (Calbiochem) [24], and 0.75 *μ*M IWR-1-endo (kindly provided by Dr. Lawrence Lum, University of Texas, Southwestern Medical Center) [25]. N2B27 medium was changed every other day. On the 6th day, EGFP-positive cells (Sox1 positive cells) were observed by using a fluorescent microscope (Leica DMIRB, Leica Microsystems). For production of neurons, Sox1-positive cells were replated on PDL-laminin-coated wells in N2B27 medium containing EGF and FGF-2. N2B27 medium was changed every 3-4 days [6].

### 2.3. FACS Analysis

To analyze 46C ES cell-derived Sox1-GFP-positive neural precursors, 46C cells were differentiated for 6 days in N2B27 medium with MeBIO or BIO and DMSO or IWR. The cells were then trypsinized and neutralized with serum containing medium. After centrifugation, cells were washed two times with PBS and resuspended in PBS. Resuspended cells were analyzed using a FACScan. For data analysis, FACSDiva software was used.

### 2.4. Transfection and Luciferase Assay

Oct4 promoter-EGFP ES cells were stably transfected with TOP-mCherry and Axin2-promoter-mCherry using Amaxa nucleofector according to the manufacturer's instructions. Stably transfected clones were selected in media containing 800 *μ*g/mL of G418 (Gibco).

To measure Wnt signaling activity, pTOP-Flash plasmid and pRL-TK plasmid were transfected into mES cells. Transfected ES cells were cultured in the presence or absence of LIF for 48 h. To measure Sox1 promoter activity under nondifferentiation and neuronal differentiation conditions, pGL3-mSox1 promoter plasmid and pRL-TK plasmid were transfected into 46C cells, which were cultured in mES medium or N2B27 medium with or without 0.75 *μ*M BIO (Calbiochem). Luciferase activity was measured by Dual-Luciferase reporter assay (Promega).

### 2.5. Reverse Transcriptase-PCR

Examination of gene expression during neuronal differentiation was carried out by RT-PCR. Total RNA was isolated using TRIzol reagent (Invitrogen) according to the manufacturer's protocol. cDNA was synthesized from total RNA (1 *μ*g) in a total reaction volume of 20 *μ*L using ImProm-II Reverse Transcriptase (Promega) with random primers. cDNA was amplified under the following conditions: 94°C for 2 min, followed by 20, 25, or 30 cycles of 94°C for 45 s, 58°C for 45 s, and 72°C for 50 s.

### 2.6. Immunofluorescence Analysis

Neuronally differentiated 46C ES cells for 15 days were fixed with 4% paraformaldehyde at room temperature for 20 min and then permeabilized for 20 min with permeabilization solution. Cells were incubated with anti-MapII (Chemicon) primary antibody for 1 h. After washing 10 times with PBST (0.05% Tween 20, 10 mM Na_2_HPO_4_, 2 mM KH_2_PO_4_, 2.7 mM KCl, and 137 mM NaCl), cells were incubated with Alexa Fluor 488 (Invitrogen) and DAPI (KPL Inc) for 1 h in the dark to stain the nucleus, followed by washing again five times with PBST. Wells were mounted in a drop of Mount Medium (KPL Inc). Signal was visualized by fluorescence using a microscope (Leica DMIRB, Leica Microsystems).

### 2.7. Western Blot Analysis

Mouse ES cells were seeded in 6-well plates and differentiated in ES medium or N2B27 medium without LIF. mES cells were washed with phosphate-buffered saline (PBS) and then lysed in RIPA buffer (25 mM Tris-HCl at pH 8.0, 150 mM NaCl, 10% glycerol, 1% Igepal CA-630, 0.25% deoxycholic acid, 2 mM EDTA, 1 mM NaF, and 50 mM glycerophosphate) on ice for 30 min. Lysates were cleared by centrifugation at 12,000 rpm for 10 min, after which supernatants were collected. Bradford (Bio-Rad) assay was used to determine the protein concentration. Equal amounts of protein were boiled and separated by SDS-polyacrylamide electrophoresis gels and transferred to a PVDF membrane (Pall Corporation). Blots were then incubated with antiactive-*β*-catenin (Millipore) or anti*β*-actin (Santa Cruz Biotechnology) antibody. Immunocomplexes were visualized using an enhanced Chemiluminescence kit (Elpis Biotech).

### 2.8. Chromatin Immunoprecipitation (ChIP) Assay

Cells were cross-linked with 1% formaldehyde (Sigma) at room temperature for 10 min and then incubated with 0.125 M glycine for 5 min with gentle shaking. Cells were then washed twice with PBS before harvesting. Cells resuspended with hypotonic buffer (10 mM Hepes-KOH, pH 7.8, 10 mM KCl, and 1.5 mM MgCl_2_) were swollen on ice for 10 min and then passed through a 26.5 gauge needle six times. After centrifugation at top speed for 5 min at 4°C, pellets were incubated with nuclei lysis buffer (1% SDS, 50 mM Tris-HCl, pH 8.0, and 10 mM EDTA) for 10 min on ice with occasional vortexing. Chromatin was sheared to an average length size of 0.2~1 kb by sonication on ice. Supernatant was collected by centrifugation (13,000 rpm, 10 min, 4°C), and the supernatant concentration was determined by a spectrophotometer. The appropriate volume of chromatin was diluted 1 : 10 in ChIP dilution buffer (0.01% SDS, 20 mM Tris-HCl, pH 8.0, 167 mM NaCl, 1.2 mM EDTA, and 1.1% Triton X-100), after which preclearing was performed at 4°C for 2 h with 10 *μ*L of protein A/G plus-agarose beads (Santa Cruz Biotechnology). For immunoprecipitation, goat, rabbit-IgG (Bethyl), anti*β*-catenin (BD biosciences), anti-TCF3 (Santa Cruz), and anti-TCF1 (cell signaling) antibodies were administered at 4°C overnight. Immunoprecipitated chromatins were eluted, after which reverse cross-linking was carried out by addition of 0.3 M NaCl at 65°C overnight. Following phenol-chloroform extraction and ethanol precipitation, DNA was dissolved in 50 *μ*L of TE buffer (10 mM Tris-HCl, pH 8.0, and 1 mM EDTA). Sox1-1 primers (Forward: 5′-AGTTCAGCCCTGAGTGAC-3′ and Reverse: 5′-TGGGTGCCTAGCGGAGAG-3′), Sox1-2 primers (Forward: 5′-TGGTCTGATCCCAAGTAG-3′ and Reverse: 5′-TTTCTGAAGCGATTCTCC-3′), and Axin primers (Forward: 5′-TAACGCGGGAGCTGAGTGTG-3′ and Reverse: 5′-AAATCCATCGCGAACGGCTG-3′) were used for PCR.

## 3. Results

### 3.1. Increase in Wnt/*β*-Catenin Signaling Activity during Neural Differentiation

**Figure 1:**
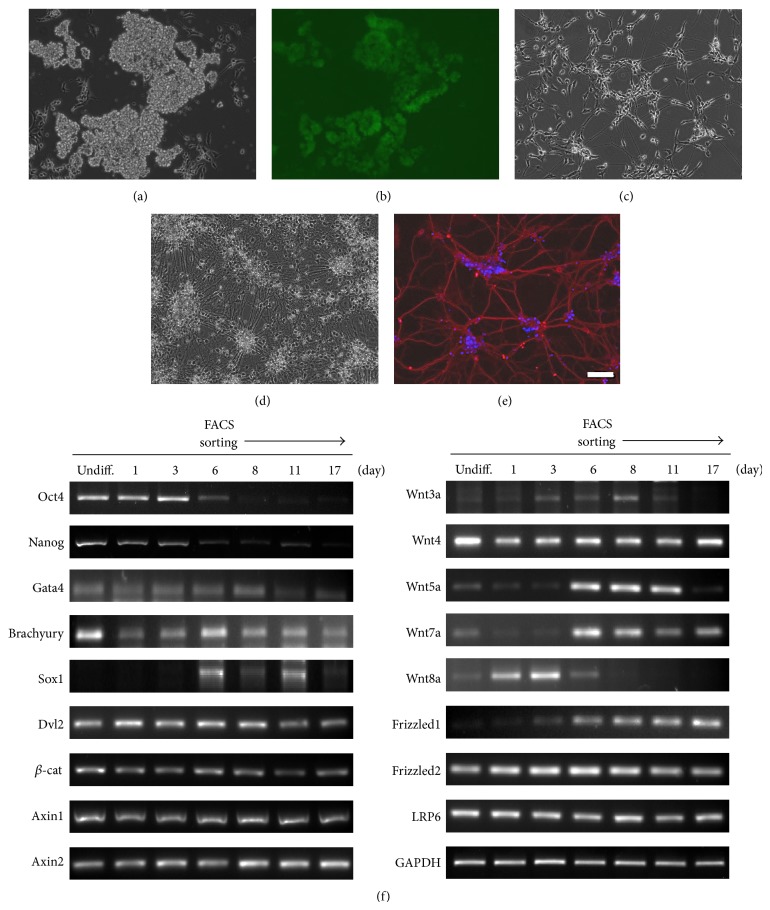
Neuronal differentiation of mouse embryonic stem cells and expression of Wnt and Wnt component genes. (a)–(e) Using N2B27 medium, 46C ES cells (*Sox1*-promoter-GFP) were differentiated to neuronal cells for 14 days. GFP in 46C ES cells was expressed during neuronal differentiation for 6 days ((a) phase contrast, (b) Sox1-GFP), and neurite outgrowth increased during neuronal differentiation for 8 days (c) and 14 days ((d) phase contrast, (e) MAPII (red), DAPI (blue)). (f) RT-PCR results of stem cell marker, Wnt, and Wnt signaling components during neuronal differentiation. During neuronal differentiation, RNA levels of many Wnt and Wnt signaling components were changed. Scale bars, 50 *μ*m.

To study the role of Wnt signaling during neural differentiation, we used 46C mouse embryonic stem (ES) cells (EGFP was substituted into the ORF of the* Sox1* gene) with the monolayer neural differentiation method. Upon being cultured in N2B27 medium, 46C ES cells started to express EGFP from day 4, and outgrowth of neurites was detected from day 8 after replating (Figures 1(a)–1(e)) [5]. To examine the expression patterns of Wnt components during differentiation, we performed RT-PCR analysis using specific primers. RT-PCR data showed reduced expression of stemness markers (*Oct4* and* Nanog*) and increased expression of an ectoderm marker (*Sox1*) during neuronal differentiation. Expression levels of many Wnts, Wnt receptors, Wnt coreceptors, and Wnts components also changed during differentiation (Figure 1(f)). These data suggest that Wnt signaling might play important roles in self-renewal or neuronal differentiation, although its exact functions are unknown.

**Figure 2:**
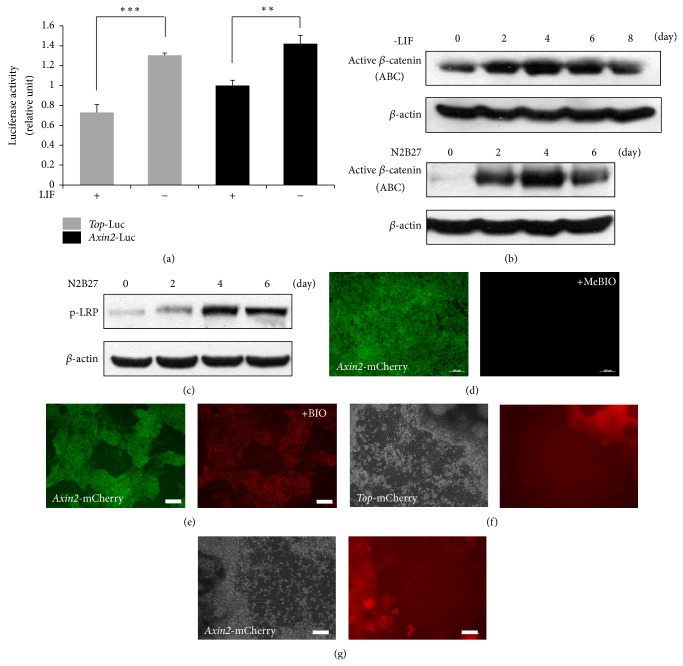
Increase in canonical Wnt signaling during neural differentiation. (a) Both TOP and* Axin2* promoter luciferase activities in E14 ES cells were induced at 48 h after LIF removal. (b) Western blot analysis using ABC (active *β*-catenin) and *β*-actin antibodies. Active *β*-catenin level was the highest on day 4 of neural differentiation. (c) p-LRP level was the highest on day 4 of neural differentiation. (d)-(e) In* Oct4-*Gip/Ax2P-mCherry cells, only GFP expression was detected. mCherry expression was increased after 24 h addition of BIO (0.75 *μ*M). (f)-(g) mCherry expression increased in the neural precursor region of TOP-mCherry ((d) and (e)) and* Ax2*p-mCherry ((f) and (g)) stable cell lines during neuronal differentiation. Stable cell lines were cultured in N2B27 medium for 14 days. Scale bars, 100 *μ*m.

Next, we asked whether Wnt signaling activity can be regulated under self-renewal and differentiation conditions. To this end, we measured endogenous Wnt/*β*-catenin signaling activity under self-renewal and differentiation conditions using TOP and Wnt target genes* Axin2* promoters (hereafter called Ax2P)-driven reporter construct that contain responsive TCF binding elements [26, 27]. Notably, reporter activities were low in undifferentiated mouse ES cells, whereas it was increased under absence of LIF conditions (Figure 2(a)). To monitor the change status of Wnt signaling during neural differentiation, we established reporter ES cell lines (Oct4-Gip/TOP or Ax2P-mCherry). As expected, we observed only GFP expression but not mCherry expression in self-renewal condition due to low Wnt activity (Figure 2(d)). As shown in Figure 2(e), addition of BIO, GSK3*β* inhibitor, enhanced expression of mCherry, indicating that this cell line reflects Wnt/*β*-catenin signaling. After stable cells were cultured in N2B27 medium for neural differentiation, expression of mCherry was significantly increased in neural precursor cells, whereas it was decreased in fully differentiated neurons (Figures 2(f)-2(g)). Consistently, the active *β*-catenin was more abundant during differentiation induced by the removal of LIF or N2B27 medium, which induces embryonic stem cells differentiation toward neuronal lineage, reaching a maximum on day 4 (Figure 2(b)). LRP6 phosphorylation on S1490, used an indicator for initial activation of Wnt/*β*-catenin signaling, was increased during neural differentiation (Figure 2(c)). Taken together, these data suggest that Wnt/*β*-catenin signaling is regulated during ES cell differentiation and neural precursor differentiation.

### 3.2. Treatment of BIO from Days 4 to 6 Enhances Neural Differentiation

**Figure 3:**
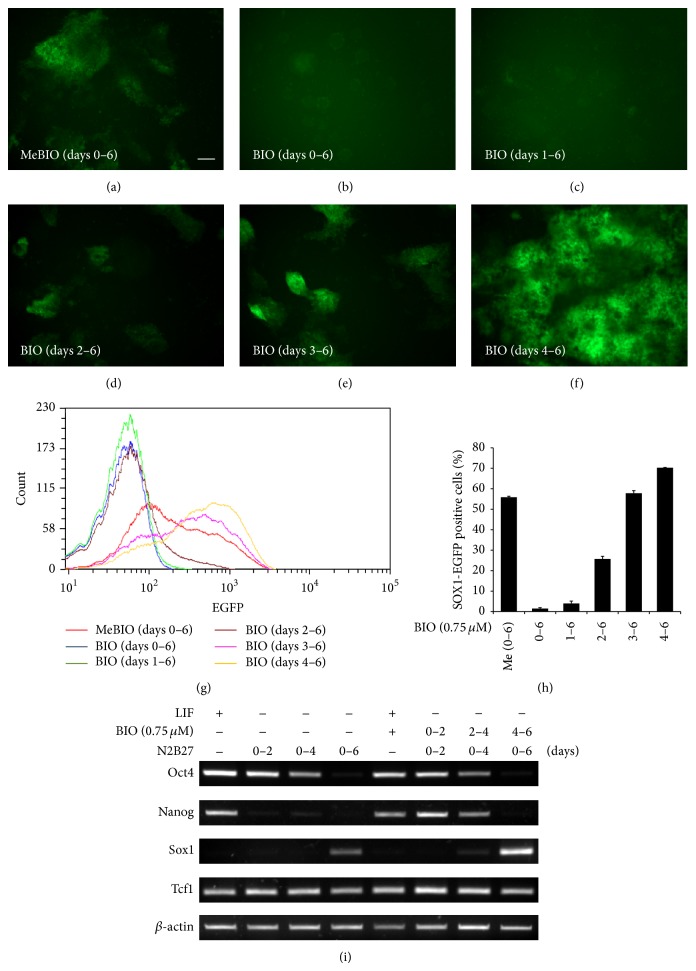
Increase in neural differentiation of precursor cells by GSK3*β* inhibitor (BIO) during days 4 to 6 in N2B27 medium. (a)–(f) 46C ES cells were cultured in N2B27 medium for 6 days. GFP expression was elevated by BIO treatment (0.75 *μ*M) from days 4 to 6, whereas GFP expression was reduced by BIO treatment (0.75 *μ*M) from days 0 to 3. (g) FACS analysis of* Sox1*-GFP expression during monolayer differentiation in N2B27 medium. (h) Proportions of* Sox1*-GFP expressing cells as determined by FACS. (i) RT-PCR analysis showed that Nanog and Sox1 expression were elevated by BIO treatment (0.75 *μ*M) under nondifferentiation and neural differentiation conditions, respectively. Scale bars, 100 *μ*m.

Based on data described above (Figures 1 and 2), we asked whether time window of Wnt/*β*-catenin signaling affects neural differentiation. For this, we sequentially treated with BIO (GSK3*β* inhibitor) for indicated duration as described in Figure 3 [24]. Addition of BIO in 46C ES cells from day 0 to day 6 completely diminished EGFP expression compared to MeBIO treatment, a control analog of BIO which displays minimal activity against GSK3*β* (Figures 3(a) and 3(b)). These results were corroborated by FACS analysis (Figures 3(g) and 3(h)). Similar to report that activation of canonical Wnt signaling by GSK3*β* inhibition maintains pluripotency of ES cell, we found that BIO treatment could enhance the expression of stemness marker gene such as* Nanog* under differentiation conditions (Figure 3(i)) [18–20, 28]. On the other hand, treatment with BIO from day 4 to day 6 surprisingly increased EGFP intensity and EGFP-positive cells (Figures 3(f)–3(h)). To test whether or not the increase in EGFP expression by BIO can be attributed to actual neural differentiation, we compared the mRNA expression levels of marker genes in cells treated or untreated with BIO. We observed that the expression* Sox1* mRNA was increased in ES cells after BIO treatment from day 4 compared to untreated cells (Figure 3(i)). These results imply that activation of Wnt/*β*-catenin signaling for different time periods has differential effects on neural differentiation of mES cells.

### 3.3. Transient Activation of Wnt/*β*-Catenin Signaling Enhances Neuronal Differentiation

**Figure 4:**
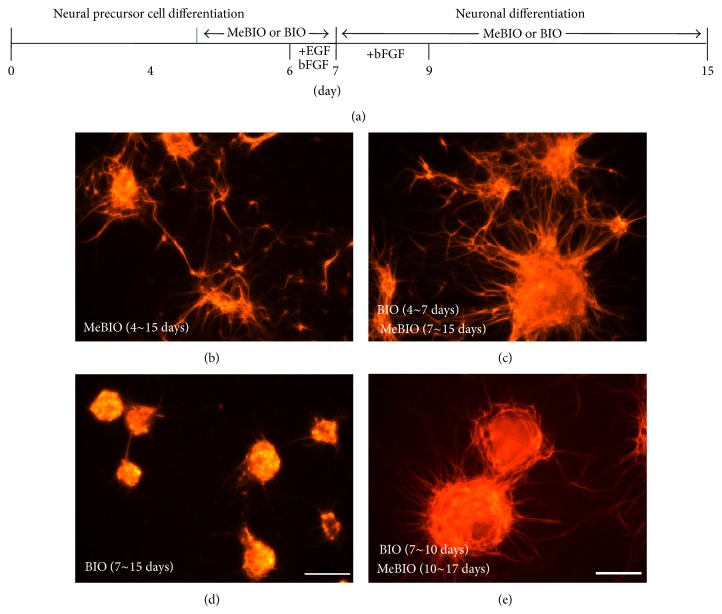
Inhibition of neurite formation during late neuronal differentiation by GSK3*β* inhibitor. (a) Diagram of MeBIO (0.75 *μ*M) or BIO (0.75 *μ*M) treatment during late neuronal differentiation of 46C ES cells. (b)–(e) Cells cultured according to the diagram for 15 days were stained with MAPII antibody. Neurite formation was elevated by BIO treatment from days 4 to 7 (c), whereas it was reduced by BIO treatment from days 4 to 15 (d). (e) Cells were cultured with BIO from days 7 to 10 and cultured with MeBIO for 7 days after replating, followed by staining with MAPII antibody. Scale bars, 100 *μ*m.

The finding that the activation of Wnt signaling differentially regulates neuronal differentiation of ES cells promotes us to define the optimal condition for neuronal differentiation. To this end, we treated with Me-BIO or BIO from 4 to 7 days during neural differentiation, followed by treatment again with Me-BIO or BIO from 8 to 15 days after replating on PDL-laminin-coated plates (Figure 4(a)). Neurite formation induced by BIO from days 4 to 7 was remarkably enhanced compared to MeBIO treatment (Figures 4(b) and 4(c)). In contrast, neurite formation was completely abolished by BIO treatment from days 7 to 15 (Figure 4(d)). Next, we checked whether or not neurite formation derived from precursor cells could be recovered by withdrawing of BIO during neuronal differentiation. We treated with BIO into precursor cells for 3 days after replating, and then cells were cultured in the absence of BIO for 7 days more during neuronal differentiation. Interestingly, neurite formation of Map2-positive cells was restored under BIO-deficient conditions (Figure 4(e)). These data indicate that transient activation of Wnt/*β*-catenin signaling during neural precursor formation may be beneficial for optimal neuronal differentiation.

### 3.4. IWR-1-Endo Treatment from Days 4 to 6 Suppresses Differentiation of Neural Precursor Cells

**Figure 5:**
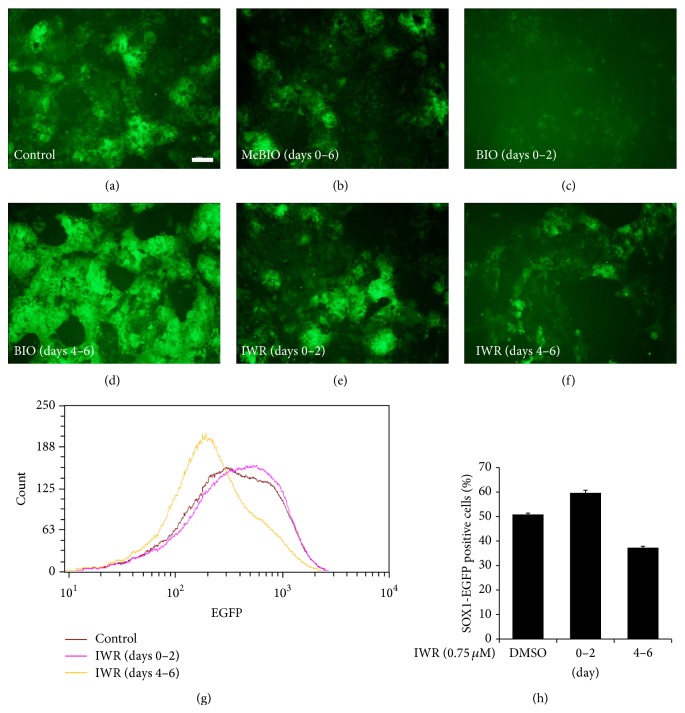
Decrease in neural differentiation of precursor cells by an Axin stabilizer (IWR-1-endo) from days 4 to 6 in N2B27 medium. (a)–(f) GFP expression of 46C cells was elevated by BIO treatment (0.75 *μ*M) from days 4 to 6, whereas it was reduced by IWR-1-endo (0.75 *μ*M) treatment from days 4 to 6. Cells were cultured in N2B27 medium for 6 days. (g) FACS analysis of* Sox1*-GFP expression during monolayer differentiation in N2B27 medium. (h) Proportions of* Sox1*-GFP expressing cells as determined by FACS. Scale bars, 100 *μ*m.

As GSK3*β* has been known to be involved in multiple signaling pathways in addition to Wnt/*β*-catenin signaling, we elucidated neural differentiation efficiency by using a specific inhibitor IWR-1-endo (Axin stabilizer) [25, 29]. Similar to data shown in Figure 3, neural differentiation of ES cells was reduced by treatment with BIO from days 0 to 2, whereas it was increased from days 4 to 6 (Figures 5(a)–5(d)). On the contrary, treatment of 46C ES cells with IWR-1-endo from day 4 to day 6 resulted in a significant reduction in neural differentiation, whereas Wnt signaling inhibition by IWR-1-endo from days 0 to 2 caused a slight increase in differentiation (Figures 5(e), 5(f), and 5(h)). These results were further corroborated by FACS analysis of EGFP expression (Figures 5(g) and 5(h)). Based on these data, we conclude that activation of Wnt/*β*-catenin signaling on days 4 to 6 is essential for the proper formation of neural precursor cells.

### 3.5. The Expression of Sox1 Is Enhanced by Direct Binding of *β*-Catenin-TCF1 to the Promoter during Neural Differentiation

**Figure 6:**
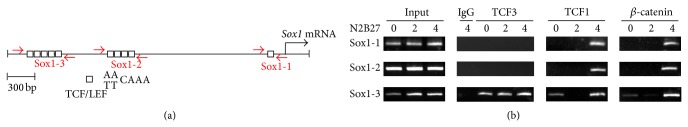
Increased Wnt/*β*-catenin signaling directly regulates Sox1 promoter activity during neural differentiation. (a) Schematic representation of binding sites for TCF/LEF transcription factors in a 3 kb promoter region of the* Sox1* gene. Three pairs of PCR primers were designed based on potential TCF/LEF binding sites. (b) ChIP analysis of the mouse* Sox1* promoter with *β*-catenin, TCF1, or TCF3 antibodies. *β*-catenin and TCF1 bound to the* Sox1* promoter from day 4 during neural differentiation.

We found that BIO enhances* Sox1* mRNA expression during neural differentiation (Figure 3(i)). Therefore, we then examined whether *β*-catenin directly could be occupied at promoter of* Sox1* gene during neural differentiation. Interestingly, several conserved putative TCF/LEF-binding sites were present in a 3 kb promoter region of both human and mouse* Sox1* gene (Figure 6(a)). In order to verify this hypothesis that the complex of *β*-catenin and TCF/LEF transcription factors could bind to these conserved binding sites, we carried out chromatin immunoprecipitation (ChIP) analysis using antibodies specific for *β*-catenin, TCF1, and TCF3. Notably, *β*-catenin and TCF1 did not access to Sox1-1 and Sox1-2 region under nondifferentiation and early differentiation conditions, whereas they associated with all three potential binding sites from day 4 during monolayer differentiation. In addition, we found weak binding of *β*-catenin and TCF1 to Sox1-3 region in self-renewal stem cells. This weak *β*-catenin-TCF1 binding on Sox1-3 at day 0 may have a rheostatic role in transcriptionally controlling expression of* Sox1* gene during differentiation. Sox1-3 region is a distal control element located around 3 kb from the transcription initiation site. Therefore, we expect that weak interaction of *β*-catenin-TCF1 keeps a ready state to easily activate genes. On the other hand, TCF3 occupancy, a known repressor protein for Wnt signaling, was not changed during differentiation (Figure 6(b)). These data indicate that *β*-catenin/TCF1 complex activates* Sox1* promoter through direct binding to the conserved TCF binding sites during the late stage of neural precursor differentiation.

## 4. Discussion

Here we showed that the expression of various Wnt signaling components is dynamically changed during neuronal differentiation (Figure 1), but the biological meaning of these changes and regulation of the expression of these genes are largely unknown. However, overall Wnt/*β*-catenin signaling activity in undifferentiated ES cells seems to be low compared to differentiated stage (Figure 2). Although it has been shown that activation of Wnt/*β*-catenin signaling by the treatment of BIO or Wnt3a enhances stemness of ES cells [18, 19, 30], these findings are not contradictory to our findings. Our data suggest that an increase of Wnt/*β*-catenin signaling is necessary (Figure 5) but not sufficient to induce neuronal differentiation of ES cells. It may be possible that increase of Wnt/*β*-catenin signaling than endogenous level in undifferentiated state is sufficient to enhance stemness, but other unknown changes during differentiation along with high level of Wnt/*β*-catenin signaling may be required for proper neuronal differentiation of ES cells.

Tsao et al. [31] showed that Sox1 acts as a tumor suppressor by inhibiting TCF-responsive transcriptional activity in hepatocellular carcinoma. Interestingly, our ChIP analysis showed that *β*-catenin-TCF complex could regulate the expression of Sox1 in the middle of neuronal differentiation periods (Figure 6). Although further study would be necessary to test the possibility, increase of Wnt/*β*-catenin signaling induced Sox1 until generation of neural precursors and then increased level of Sox1 suppresses Wnt/*β*-catenin signaling during late neuronal differentiation period.

We identified that treatment of BIO at specific time points during differentiation can enhance neuronal differentiation (Figure 3). Treatment of BIO from the beginning of differentiation as well as continuous activation of Wnt/*β*-catenin signaling until late neuronal differentiation stage blocked neuronal differentiation, which might be due to enhancing self-renewal of ES cells and inhibitory effect of Wnt/*β*-catenin signaling on late neuronal differentiation period, respectively, as shown before [18, 19, 23]. Our findings could be useful information in therapeutic point of view using stem cells, since the treatment of Wnt/*β*-catenin signaling activator along with small molecules regulating other signaling pathways will further enhance neuronal differentiation.
